# How do Midwives and Physicians Discuss Childhood Vaccination with Parents? 

**DOI:** 10.3390/jcm2040242

**Published:** 2013-11-04

**Authors:** Eve Dubé, Maryline Vivion, Chantal Sauvageau, Arnaud Gagneur, Raymonde Gagnon, Maryse Guay

**Affiliations:** 1 Quebec National Institute of Public Health, Quebec G1E 7G9, Canada; E-Mails: maryline.vivion@inspq.qc.ca (M.V.); chantal.sauvageau@inspq.qc.ca (C.S.); maryse.guay.agence16@ssss.gouv.qc.ca (M.G.); 2 Laval University Hospital Research Center, Quebec G1V 4G5, Canada; 3 Department of Pediatrics, Étienne Le-Bel Research Center of Sherbrooke University Hospital, Quebec J1H 5N4, Canada; E-Mail: arnaud.gagneur@usherbrooke.ca; 4 University at Trois-Rivières, Quebec G9A 5H7, Canada; E-Mail: raymonde.gagnon@uqtr.ca

**Keywords:** Canada, vaccination, decision-making, physicians, midwives, knowledge, attitudes, practices

## Abstract

Even if vaccination is often described as one of the great achievements of public health, results of recent studies have shown that parental acceptance of vaccination is eroding. Health providers’ knowledge and attitudes about vaccines are important determinants of their own vaccine uptake, their intention to recommend vaccines to patients and the vaccine uptake of their patients. The purpose of this article is to compare how midwives and physicians address vaccination with parents during pregnancy and in postpartum visits. Thirty semi-structured interviews were conducted with midwives and physicians practicing in the province of Quebec, Canada. Results of our analysis have shown that physicians adopt an “education-information” stance when discussing vaccination with parents in the attempt to “convince” parents to vaccinate. In contrast, midwives adopted a neutral stance and gave information on the pros and cons of vaccination to parents while leaving the final decision up to them. Findings of this study highlight the fact that physicians and midwives have different views regarding their role and responsibilities concerning vaccination. It may be that neither of these approaches is optimal in promoting vaccination uptake.

## 1. Introduction

The decline in vaccine-preventable diseases, along with the increasing number of vaccines offered in child schedules, have challenged the acceptability of vaccination for parents [1]. Parents are often uncertain about the risks and benefits of vaccination and many studies have shown that a significant proportion of parents have doubts regarding vaccination [2,3,4,5]. Recent outbreaks of infectious diseases among unvaccinated individuals also suggest that concerns regarding vaccination are widespread in Canada and in other countries [6,7,8]. Many studies have shown the crucial role that health providers play in the success of vaccination programs, as their recommendations are one of the key determinants of parents’ decision to have their child vaccinated [9,10,11,12,13,14]. 

In Quebec, Canada, vaccines against 13 diseases are offered free of charge to parents through the universal provincial immunization program. Vaccination is not mandatory and parents are free to accept or decline vaccination for their children. Children vaccination schedules involve five vaccination visits when the child is aged 2, 4, 6, 12 and 18 months old. Most vaccines are administered by nurses in public community health services (CLSC) or by physicians and/or nurses in private offices. 

Previous studies have also shown that the decision-making process about vaccination of one’s child may begin during pregnancy [4,15,16,17]. Pregnant women in Quebec are usually treated by family physicians or obstetricians-gynecologists. Recently, pregnant women have also begun to receive care from midwives. Midwifery was authorized by law in 1999 in Quebec [18]. It includes prenatal care for normal pregnancies (*i.e.*, pregnancies without any special medical conditions), birth delivery and 6-weeks postpartum monitoring of mothers and newborns. Midwifery practice take place in *Maisons de naissance* which are locations that welcome pregnant women and their families. They are located in houses in the community, distinct from both the home and the hospital center, but part of the public health system. They are designed to allow for a reasonable number of births each year that preserve an intimate, family and human character [19]. In 2009, 1.9% of births in Quebec were assisted by midwives and the objective of the Quebec Ministry of Health is to increase access to these professionals so that up to 10% of Quebec pregnant women have the assistance of a midwife by 2018 [20]. Since 2008, midwives have also been authorized to administer some vaccines (hepatitis B, measles, mumps and rubella (MMR) vaccine) as well as immunoglobulin against hepatitis B [21]. However, results of two Canadian studies have shown that births assisted by midwives were associated with incomplete vaccination status of the child [22,23].

The aim of this study is to compare how midwives and physicians address vaccination with parents during pregnancy and in postpartum visits so as to better understand their potential influence on parents’ decisions about vaccination.

## 2. Methods

This study is based on in-depth interviews conducted with physicians who assist births and with midwives. Interviews with midwives were conducted during the summer and fall of 2010 and interviews with physicians, during the fall of 2011. 

### 2.1. Recruitment of Participants and Data Collection

In Quebec, there are approximately 130 midwives registered with the *Ordre des sages-femmes du Québec* [24]. Purposive sampling was used to recruit midwives from all 11 *maisons de naissance* of the province. 

The physician sample was constituted intentionally. A convenience sample of physicians who assist births or follow newborn babies was constituted by mailing invitations to participate to a random sample of physicians listed on the websites of five health agencies of the province. 

Participation by midwives and physicians was voluntary, and a small monetary compensation was given to participants. The ethics approval for the study was obtained from the principal author’s institution. All participants provided written informed consent.

Interviews were conducted using a semi-structured guide (Table 1). The guide was designed to elicit information about: Academic courses and work experience, practices around pregnancy and birth, approaches to health and prevention and, more specifically, approaches to vaccination. Interviews were loosely conducted and, in an iterative process, the interview guide was adjusted throughout data collection. All interviews were conducted by a research professional trained in anthropology (Maryline Vivion). 

The sample was constituted purposefully; interviews were conducted with participants having different socioprofessionals characteristics (age, number of year of practice, training, *etc.*) in order to include different perspectives regarding the main themes of the study. Participants were recruited and interviews were conducted until data saturation was reached within each group of professionals, that is, when no new ideas emerged during the interviews for the main themes [25]. 

### 2.2. Data Analysis

All interviews were audiotaped and transcribed verbatim. A content analysis of transcribed interviews was done using N’Vivo 9.0. All transcribed interviews were read by three authors (Eve Dubé, Chantal Sauvageau and Maryline Vivion). Data codification was performed by Maryline Vivion. Data were organized into main coded themes which followed the interview guide, with a particular focus on vaccination-related themes. After coding a few of the verbatim texts, the coding tree was discussed by the authors (Eve Dubé and Maryline Vivion) and adjusted. Ambiguous verbatim texts were discussed between authors (Eve Dubé and Maryline Vivion).

Thirty interviews were conducted: 17 with midwives and 13 with physicians. Interviews lasted on average 60 min with midwives and 30 min with physicians. Interviews with physicians were shorter as they generally had fewer comments on vaccination than midwifes. In addition, as all interviews were conducted at the providers’ office, duration of the interviews was similar to the length of consultation with patients of physicians and midwives. Mean number of years of practice was 9 years for midwives and 16 years for physicians. Physicians and midwives were practicing in different regions (urban and rural) of the province of Quebec. All interviews were conducted in French. Quotes provided in the following sections were selected on the basis of their clear representation of the key themes. Quotes were translated into English and submitted to a back-translation to French to ensure that their meaning was maintained.

**Table 1:** Semi-structured guide for the interviews with physician and midwives.

Main themes	Examples of questions
Academic and professional background	Why did you choose to be a midwife?/to assists births (for physicians)?
Perception about their professional role during prenatal follow up	Can you describe your approach to pregnancy? How do you present prenatal tests to families?
Perception about their professional role during delivery	Can you describe your role during delivery?
Perception about their professional role during postnatal follow up	Can you describe your role after delivery? What kind of support do you offer to families after birth (e.g., breastfeeding)?
Opinion regarding their role in health prevention	Do you consider health promotion an important part of your counseling with pregnant women? Why? From your point of view, what is the most important thing to do for disease prevention? From your point of views, what are vaccination’s pros and cons?
Opinion regarding vaccination	How (and when) do you discuss vaccination with parents? Do you consider vaccination an important part of your work? Do you administer vaccines yourself? How do you manage parents unsure about vaccination?

## 3. Results

### 3.1. Health Promotion Roles and Practices

Both midwives and physicians considered that they have an important role in health promotion and disease prevention. However, how they actualize this role differs greatly. 

Midwifery philosophy promotes the respect of the normal process of pregnancy, empowerment of mothers and families, informed choice and a personalized approach to health. Indeed, midwives emphasized the importance of parents taking decisions by themselves, and saw their role more as one of providing information. Physicians more often considered their role as that of an “advisor” or an “educator”. Thus, in health promotion, they were more prescriptive in telling parents what they ought or ought not to do. 

All interviewed midwives viewed their particular approach in opposition to biomedicine and most were critical of biomedical obstetric practices. Midwives stressed their openness to alternative medicine practices and their emphasis on the natural continuum of pregnancy and birth. Interviewed midwives also stressed their personalized approach to health promotion in pregnancy. In contrast, physicians referred more systematically to a list of themes to be addressed with patients. 

Length of consultation varied greatly between midwives and physicians, with approximately 1 h of consultation each month for midwives compared to 15 min each month for physicians. The fact that midwives’ consultations last longer was perceived by midwives as facilitating health promotion counseling with parents. Differences in the perceptions of midwives and physicians as to their role in health promotion are also reflected in their practical approach to prenatal diagnosis tests. Many prenatal tests are offered free of charge to Quebec pregnant women (e.g., echography, screening for diabetes or trisomy 21). Physicians and midwives have different ways of presenting these tests to parents. Physicians tend to prescribe systematically all available “routine” tests to pregnant women. It is important to note, however, that the approach of physicians was more nuanced regarding genetic screening tests like the trisomy 21 test. The pros and cons of genetic tests were always discussed with patients and, generally, patients’ decisions were treated with respect. 

In contrast, midwives advocate for a “judicious use” of technology and, with an emphasis on parent empowerment, prefer to give information and leave the decisions on whether or not to undergo the test to parents. Some of the interviewed midwives were also very critical of the routine and systematic use of testing by physicians. 

### 3.2. Physician and Midwife Knowledge and Opinions about Vaccination

When asked what factors have contributed to the decrease in infectious diseases, vaccination was mentioned by all interviewed physicians, along with improved hygiene. In comparison, only three midwives spontaneously answered vaccination. Most midwives attributed the decrease in infectious diseases to hygiene, better food and breastfeeding. When asked about the pros and cons of vaccination, physician and midwife responses were different. All physicians were highly supportive of vaccination while midwife responses indicated doubts regarding the usefulness and safety of vaccination. Among the “pros” of vaccination, the decline or eradication of infectious diseases was mentioned by half of physicians (6/13) and by one out of 5 midwives (3/17). Six midwives and four physicians also talked broadly about the disease prevention offered by vaccination. The fact that vaccines are free and the efficacy and safety of vaccines were mentioned by physicians (3/13) and midwives (6/17) as other arguments in favor of vaccination.

Spontaneously, four physicians stated that there were no arguments against vaccination. The others talked about the fact that some vaccines are not included in the free universal vaccination program and thus that parents have to pay for them (5/13) or about the increasing number of vaccines in the child schedules (3/13). Three midwives also mentioned this argument. In fact, the recent inclusion of new vaccines (against varicella and rotavirus) in the Quebec national vaccination program for children raised questions among both physicians and midwives. Ten midwives considered that it was not important to vaccinate children against varicella and four, against rotavirus. Some physicians also partially shared this opinion, describing the varicella vaccine (4/13) and rotavirus vaccine (5/13) as “less important vaccines”. However, six physicians clearly stated that all vaccines were important and four were highly supportive of varicella vaccination. One of them showed a picture of a child with complications from varicella as an argument used to convince parents to have their child vaccinated.

Midwives also noted adverse events after vaccination (9/17), the fact that long-term efficacy of vaccines was unknown or deficient (11/17), the lack of “unbiased” information regarding vaccination (9/17) and the fact that vaccines were used to prevent mild diseases (7/17). None of the interviewed physicians mentioned any of these arguments. Physicians and midwife opinions regarding the Quebec provincial vaccination program were also very divergent (Table 2). While all physicians approved of the child vaccination schedule, 12 midwives felt that childhood vaccination starts too early. Physicians were also highly supportive of combined vaccines, which reduce the number of injections for children whereas most interviewed midwives considered that combined vaccines restricted the possibility for parents to choose which antigens their child should receive. 

**Table 2:** Midwives’ and physicians’ opinions regarding some aspects of the Quebec national vaccination program.

Vaccination program aspects	Midwife quotes	Physician quotes
**Vaccination schedule** 12/17 midwives disapprove of the vaccination schedule 13/13 physicians approve of the vaccination schedule	I think making everyone follow the same vaccination schedule means that we lose some people who perhaps should be vaccinated, because of the rigidity. Some feel that it starts too young and so they won’t go, but they won’t end up going later on either. (Midwife, 15–19 years practice). Well, I think it’s too soon, I understand that you want to get the maximum immune response in children and by giving them early, you have a bigger immune response and at the same time, I think it’s too soon to be putting viruses into the body of a little human being. (Midwife, 5–9 years of practice).	The goal is to try and protect them at the point in their lives when they are most vulnerable, the more you wait, well, the period of greatest vulnerability is past, well, I mean, the younger they are, we know the risk of being affected is greater. (…) So no, I don’t think that it starts too soon. (Physician, 18 years practice).
**Use of combined vaccines** 12/17 midwives disapprove of use of combined vaccines 13/13 physicians approve of the use of combined vaccines	Personally, I have questions on the scientific level in the sense that there are so many things that we still don’t know about the interactions among viruses themselves and about what the impact may be of injecting the three viruses at the same time, in real life, are we really in contact with rubella, measles and mumps all at the same time? I don’t know, it’s not impossible, but you know, it makes you wonder. Have we taken the time to look at this? (Midwife, 5–9 years practice).	The more vaccines are combined, the fewer shots there are, (…) myself I find it to be a lot, so uh, the more vaccines are combined together, at the administrative level it’s better, and if there are no side effects, I think that if we had a vaccine to give once, one shot, that would be still better, but … or that contained all the vaccines, but I really like the combined vaccines. (Physician, 28 years practice).

Finally, all interviewed physicians considered their knowledge on vaccination as sufficient while five out of 17 midwives felt they were lacking knowledge about vaccination.

### 3.3. Physicians’ and Midwives’ Counseling on Vaccination

Usually, physicians talk about vaccination with parents during the first postnatal visit which takes place between 2 weeks and 1 month after birth. Midwives usually discuss vaccination with parents at the end of their postnatal follow-up, *i.e.*, 6 weeks after birth. All interviewed physicians stated that they systematically discuss vaccination with parents. However, three midwives told us that they did not consider vaccination a part of their practice while the others systematically or usually discussed vaccination with parents. Nine physicians stated that childhood immunizations were administered in their office. Also, seven midwives mentioned that the vaccines that they were authorized to provide were administered in their office. When vaccination was not administered in an office, physicians and midwives referred parents to the CLSC.

As for health promotion, the way that vaccination is presented to parents varied between physicians and midwives (Table 3). Most midwives considered their role as one of giving information to parents without positioning themselves either personally or professionally. As a way of encouraging parents’ informed choice regarding vaccination, interviewed midwives said that they always gave “balanced” information on vaccination. To present the “pros” of vaccination, midwives usually gave documents produced by government agencies and used books or texts produced by alternative medicine practitioners to present the “cons”. Ten midwives also stated that they present vaccination to parents as a choice. In contrast, physicians saw their role as one of explaining, educating and encouraging parents to have their children vaccinated. Few physicians said that they systematically gave written information, as most rely on their professional knowledge and experiences. Physicians also often expressed their personal, pro-vaccination opinions to patients. 

The different approaches of physicians and midwives regarding vaccination counseling are even more evident when they are asked about their reactions to parents who are opposed to the vaccination of their children. When faced with a parent who refuses to vaccinate his or her child, both physicians and midwives asked them about their reasons for refusal, so as to explore whether their choice was a thoughtful one. However, midwives said that, if they judged that the parents had good reasons not to vaccinate, they would respect this choice in order to maintain a trusting relationship with them. In contrast, physicians acknowledged being very uncomfortable with a parent’s decision to refuse vaccination. Most of them clearly positioned themselves in favor of vaccination and tried to convince parents to change their minds. 

Communication strategies used and reactions of physicians and midwives when confronted with parents opposed to vaccination are shown in Table 4.

**Table 3:** Approaches of physicians and midwives regarding vaccination counseling.

Midwife quote	Physician quote
Yes, I talk about the vaccination schedule, and yes, I talk about vaccination. The only thing I do, without creating a controversy around vaccination, is to inform them that it is a choice and so they have the possibility of not vaccinating their child or of putting off until later the start of vaccination, even if this goes against the position of public health authorities. (Midwife, 5–9 years practice).	I show my colors right away. I’m very pro-vaccine so for sure, I think that shows in the way I approach the subject. Right away, people say, okay, she’s really (laughs) (…) but I’m not at all closed to their questions, that’s not what I mean, but I’m going to start right away with a very positive vision of vaccination and then (…) as parents, it’s very reassuring and very positive to be able to protect our children so, well, people can see clearly my position. (Physician, 7 years practice).

**Table 4:** Communication strategies used and reactions of midwives and physicians when faced with parents who refuse to vaccinate or who have significant doubts regarding vaccination.

Communication Strategies	Midwife Quotes	Physician Quotes
Exploration of parents’ reasons for choosing not to vaccinate	Sometimes there are parents who are against vaccination just because they’re against everything, so, at that point, I’ll encourage them to go get information. My goal is not necessarily to make them change their mind, but to ensure that it’s really a conscious choice. (Midwife, 5–9 years practice). Yes, and sometimes we’ll have parents in front of us who are better-informed than we are about their choice. On the other hand, there will be some who make a choice not based on anything, for sure, I don’t want to let anyone go whose choice is not based on anything. I make sure that their choices are conscious and as well, I try to make them realize that, at least as far as not having your child vaccinated goes, unfortunately, when you make a choice that goes against the direction most people are taking, you need to be even better-informed than when you make the same choice as most other people. For me, what I would like is that, whatever the choice is that you make, that it be a conscious choice. (Midwife, 5–9 years practice).	So then you ask a few questions, why, then you try to see a bit if their arguments are based on facts, or on anecdotes, like they are most of the time, (…) (Physician, 18 years practice). For the “Oh, no, I’m against that”, well then I’ll dig a bit, then I always tell them, I’m going to give you my speech once, the big speech once, after that I’ll ask you again, have you changed your mind? But when people come with preconceived notions often it’s difficult to get them to change their mind. (Physician, 7 years practice).
Arguments used with parents opposed to vaccination	One of them that I bring up is also the psychological state in which a parent could be, for example, if he says, well, me, I don’t want to have my children vaccinated and in the end a child gets meningitis and dies or has after-effects, *etc*., how will you feel about your choice, once your child is ill and you see the condition they are in now, will you regret not giving the vaccination when we know that getting vaccinated does not produce interminable side-effects or illnesses that the child might have and that are supposedly caused by vaccination? (Midwife, 15–19 years practice). If they decide not to vaccinate, it’s their decision. Yes, talk about the downside of not vaccinating and talk about disease prevention and also talk about knowing more about the diseases that their children can have so that they know how to react, if, for example, their child gets measles, to know the symptoms a bit better and how to prevent these diseases and what to do. (Midwife, 0–4 years practice).	Then I give them concrete examples (...) that I have seen, during practicums, cases of whooping cough, cases of meningitis, all the cases we can see, I remind them that they still exist, and that we see them, which is why it is important to get vaccinated too. (Physician, 1 year practice). I tell them, “The risk of your child having encephalitis is 1 in a million doses while the risk of having the natural disease is 1 in one thousand and the child can have after-effects. So you’re lucky because the majority of people get vaccinated, that protects your baby”. (Physician, 18 years practice).
Reactions to parents’ refusal to vaccinate their child	There are people who are really against vaccination and who we can see have really made an enlightened personal choice, it’s for sure that I will respect their choice when I know there’s been a whole process leading up to it. (Midwife, 5–9 years practice). There are people who are totally against, and I mean, I don’t have the impression or the pretention that I’m going to make them change their minds, but I just want to ensure that nevertheless they are conscious of it. (Midwife, 5–9 years practice).	I’ll note in the file that it’s been discussed, that the parents refuse vaccination despite the information that has been given, and I’ll sign like that. I mean that if I have a baby who has pneumococcal meningitis because the parents refused vaccination, and the baby has after-effects or dies, well, I’ll say, look, I told you that we could save the baby’s life, it was your choice. (Physician, 18 years practice). Well, I remember a case where effectively the child wasn’t vaccinated and had an important complication, I think it was whooping cough, the individual, well ... didn’t regret it but said, “Ha, well, maybe I should have had the child vaccinated” but I didn’t go say, “Ha, ha, ha, I told you so, eh” I didn’t insist, (...) I would never say to someone, “Well, you asked for it! You went looking for it! Too bad for you!” no, that wouldn’t be right. (Physician, 28 years practice). (…) A woman who decides not have her children vaccinated, for me, I wouldn’t keep her as a patient, because she will contaminate my children in the waiting room, with whooping cough or something, so usually I will tell her, “Go find another doctor”. What saves people is antibiotics, vaccines and drinking water, so it’s very important for me. (Physician, 35 years practice).

## 4. Discussion

Despite being considered to be one of the most successful public health measures [16,26,27], vaccination is perceived as unsafe and unnecessary by a growing number of parents. Recent outbreaks of vaccine-preventable diseases in several parts of the developed world have shown the devastating consequences of under- or non-vaccination [28]. In Quebec, vaccination is voluntary and there are no vaccine mandates. Around 80% of two-year-old children in Quebec have received all recommended vaccines [29]. This high rate of childhood vaccination coverage indicates that vaccination remains widely accepted among Quebec parents. However, results of recent surveys have indicated that a significant proportion of them have doubts and concerns about vaccination [30,31]. In 2011, the province of Quebec experienced the largest epidemic of measles in the Americas since this disease was declared eliminated in 2002 [32]. When parents have doubts regarding vaccination, health professionals remains the most trusted source of information [30]. 

The results of this qualitative study indicate that physicians and midwives have different views regarding their role and responsibilities concerning vaccination. As has already been shown by results of large quantitative studies conducted among Canadian clinicians [33,34], physicians interviewed in this study were strong supporters of childhood vaccination programs. They saw their role as one of promoting vaccination by educating and encouraging parents to vaccinate their children. To convince parents to vaccinate, they expressed their personal opinions and used their professional experience of vaccine-preventable diseases. A parent’s decision to question or refuse vaccination was also very challenging for interviewed physicians. Indeed, results of studies conducted in the US have shown that up to one-third of physicians would refuse to keep parents in their practice who are opposed to vaccination [35,36].

In contrast, midwives’ opinions regarding childhood vaccination were more mixed. While the majority of interviewed midwives agreed with the benefits of vaccination in preventing infectious diseases, almost all disagreed with some of the components of Quebec’s childhood vaccination program. For instance, despite the evidence showing the high risk of contracting many vaccine-preventable diseases in the first year of life (e.g., pertussis, meningitis caused by *Haemophilus influenzae* type B, pneumococcal infection), most midwives considered that vaccination begins too early in life.

Interviewed midwives saw their role as one of giving information and presenting the pros and cons of vaccination while leaving the final decision to parents. Indeed, all midwives said they respected parents’ decision to vaccinate their child or not if they judged that this decision was deliberate. Some midwives also considered that vaccination was not part of their practice and did not engage in discussion on this topic with parents. It is also important to note that, despite the fact that midwives are authorized to administer some vaccines, most *maisons des naissances* do not keep vaccines on their premises. In addition, the follow-up by midwives ends at 6 weeks post-partum, just before the first vaccination visit, planned at 8 weeks. Both these facts, along with possibly less positive attitudes regarding vaccination, could explain, at least partially, why some midwives do not feel involved in vaccination despite their strong commitment to health promotion.

The different ways physicians and midwives present vaccination to parents could also be seen as rooted in different views about informed consent and informed choice that are built into biomedical and midwifery philosophies. Medical practice is governed by a code of ethics and a medico-legal guide that present key concepts and fundamental legal principles governing medical practice [37]. The Quebec Immunization Protocol [38] stipulates that health care professionals should inform, in clear language, vaccine recipients about the risks and benefits of vaccines to be administered. Physicians also have the duty to inform all patients about vaccines recommended for them, even if the vaccines are not included in the free universal vaccination program [39]. Informed consent, in biomedicine, focuses on three components: legal, ethical, and administrative compliance [40]. Due to the fact that physicians are at risk of prosecution, legal aspects tend to take an important place in the process. 

Like physicians, midwives also have a code of ethics and a guide of practical norms by which to conduct their practice [41,42]. These are written in accordance with the midwifery philosophy which is based on a recognition of the natural process of pregnancy and birth. Informed choice and empowerment are key principles of this philosophy. Indeed, Quebec’s midwifery philosophy specifies that midwives “view decisions as a results of a process where responsibilities are shared between women, their family (as defined by women) and health professionals” and insists that midwives should “acknowledge that the final decision belongs to the woman” [19]. In contrast to the medical concept of informed consent, which often implies compliance with a higher authority, the approach of midwifery suggests that women have the power or opportunity to choose among meaningful alternatives [43]. Our study highlighted the importance of the midwife philosophy in guiding the entire practice. The informed choice is one of its principles, and midwives approach many health promotion issues, including vaccination, on the basis of this principle. While Quebec nurses and doctors must recommend vaccination to their patients [39,44], midwives consider their role as that of an information provider instead, presenting the pros and cons of vaccination to parents. 

However, for consent or choice to be informed, it is not just a question of giving relevant information and letting the patient decide what is best for him or her. It is also about the nature of the information given to the patient and about the way it is presented [45]. In this study, physicians actively promoted vaccination while midwives, in the perspective of informed choice gave information to parents about the pros and cons of vaccination. The nature of information given by physicians and midwives was also largely different. Physicians gave standard information about mild and frequent reactions after vaccination, such as fever or pain at the injection site [38]. In contrast, to illustrate the “cons” of vaccination, midwives gave parents books or texts from alternative medicine practitioners, which are very critical of vaccination and often put forward non evidence-based events attributed to vaccines, such as the onset of autoimmune diseases [46,47]. This disparity in the nature of information regarding potential adverse events after vaccination could certainly lead to different decisions among parents [1]. In addition, it may be hard for parents to get a sense of the very polarized information on vaccination [48]. 

Besides the nature of the information given to parents, the way of presenting this information could also impact parents’ decisions. Some studies have highlighted that parents find it difficult to have an open discussion about vaccination with their physician and report feeling alienated when vaccines are discussed [49]. In contrast, discussion about vaccination with alternative medicine practitioners, such as naturopaths, was perceived to be more in line with what the parents perceived to be an ideal consultation for their children than was the case for consultations with physicians [50]. In our study, physicians used a prescriptive approach by strongly encouraging parents to vaccinate, sometimes without much openness about parental concerns regarding vaccination. Clearly, some of the interviewed physicians were using a “knowledge deficit approach”, assuming that parents who were uncertain or who refused vaccination lacked knowledge and that their role was to educate them, without much consideration for their opinions and values. Instead of simply delivering standard messages on diseases or vaccines, physicians could develop more dialogue-based approaches which work with and build on the concepts that parents already use to think about vaccination [51]. 

In contrast, midwives adopted a neutral stance by not positioning themselves, either professionally or personally, for or against vaccination. This approach could be viewed as a form of disengagement by health professionals [52]. Others have highlighted that putting too much emphasis on being neutral could impede real communication between health professionals and patients [53]. Informed choice supposes that patients do take decisions by themselves. However, studies have shown that many patients prefer to delegate their decisions or to defer to the opinions of others, including their health providers [40,54,55] as a way of sharing the burden of health responsibilities [56]. In addition, no information is value-neutral. Studies [53,57] have shown that even an approach that aims at giving balanced information is often prescriptive [43]. 

As Leask and collaborators have pointed out, there are challenges to ensuring valid consent in the field of vaccination [58]. One of these challenges lies in accommodating different lay and public health views about the relative merits of vaccination in a context where risk-benefit ratios of vaccination are less apparent, as a result of the decline in vaccine-preventable diseases [59]. In addition, as for all health interventions, no vaccine is completely safe or effective and health providers have to communicate these uncertainties to patients [51]. Communicating uncertainties is a very challenging task [60] and there are many issues involved in communicating the risks and benefits of vaccination [1]. One of these is the presentation of vaccination from a top-down population-level intervention perspective to parents who are evaluating the appropriateness of vaccination in relation to their child’s particular health [51]. The inclusion of new vaccines in the childhood program to prevent diseases that could be perceived as mild (e.g., varicella or rotavirus gastroenteritis) is also challenging the communication of risks and benefits of vaccination to parents. Like parents [5,61], midwives and physicians were concerned by the increasing number of vaccines in the childhood schedules. The changes in childhood vaccination schedules and rapid developments in the field of vaccines challenged providers who have to handle a lot of vaccine-related information to be able to ensure that their knowledge is up-to-date. Many articles in the literature have stressed the importance of health providers addressing concerns of vaccine-hesitant patients in a well-managed way and authors have given their tips to providers on how to do so [62,63,64,65]. Although the approaches presented in these articles vary, they do share some common characteristics, such as the importance of maintaining a trustworthy patient-provider relationship and the importance of tailoring the communication to specific patients’ concerns and doubts. 

This study has strengths and limitations. First, as for all studies relying on qualitative interviews, social desirability bias cannot be excluded. However, the fact that interviews were conducted by a research professional from the anthropological field should have reduced this bias. Second, the sample of participants was constituted by on a voluntary basis, which could lead to a selection bias. Indeed, even if saturation of data was attained, results of this study cannot be extrapolated to all midwives and physicians working in Quebec. Qualitative researches imply a limited number of participants. However, our sample was constituted using diversification criteria, as recommended in qualitative research [66,67]. To our knowledge, this is the first study to explore the approach of Quebec midwives to vaccination using the same interview scheme to be able to compare their opinions with those of physicians. Thus, this study is exploratory in nature. Despite this limitation, as their role is crucial in sustaining the success of vaccination programs, results of this study could be useful to develop educational tools to enhance health providers’ communication about vaccination to new parents. Finally, this study reports only on the point of view of health practitioners. Patients’ opinions about the vaccination discussion with their health provider have not been assessed. The perspective of parents should be explored to have a better understanding of the impact that the discussion by providers has on parental vaccine decision-making.

## 5. Conclusions

The knowledge and attitudes about vaccines among health providers have previously been shown to be an important determinant of their own vaccine uptake, their intention to recommend the vaccine to their patients and the vaccine uptake of their patients. Results of this study indicate that physicians and midwives have different views regarding their role and responsibilities toward vaccination. Midwifes and physicians need to reflect on how they deliver information on vaccination to parents. Is it done in a paternalistic way or does it support informed parental decision-making? Are they really providing neutral, non-biased, information? Do they listen to parents’ questions and concerns about vaccination with empathy and in a nonjudgmental way? Do they discourse present evidence-based scientifically sound information on vaccination? In the context where parental acceptance of vaccines is apparently eroding, the support of health providers is essential to ensure the success of vaccination programs that rely on high level of vaccine uptake.
